# Enhanced recovery after surgery (ERAS) protocols: Impact on patient outcomes

**DOI:** 10.6026/973206300213599

**Published:** 2025-10-31

**Authors:** Pranavkumar Amrutlal Parthsarthi, Ankit Ashok Agrawal, Alpeshkumar Parmar, Manchireddy Manish, Indraj Arora, Manisha Kumari

**Affiliations:** 1 Department of General Surgery, Nootan Medical College and Research Center, Sankalchand Patel Vidyadham, Visnagar, Gujarat, India; 2 Department of General Surgery, Government Medical College, Nagpur, Maharashtra, India; 3 Department of General Surgery, Dr, N.D. Desai Faculty of Medical Sciences and Research, Dharmsinh Desai University, Nadiad-387001, Gujarat, India; 4 Department of Anesthesiology, Madha Medical College and Research Institute, Kovur, Chennai, Tamil Nadu, India; 5 Department of Oral and Maxillofacial Surgery, Hitkarini Dental College and Hospital, Hitkarini Hills, Dumna Airport Road, Jabalpur, Madhya Pradesh, India; 6 Department of Periodontics and Implantology, Hitkarini Dental College and Hospital, Hitkarini Hills, Dumna Airport Road, Jabalpur, Madhya Pradesh, India

**Keywords:** Enhanced recovery after surgery, ERAS, postoperative recovery, pain management, complication rates, hospital stay, patient outcomes, abdominal surgery and multimodal care

## Abstract

An ERAS protocols in improving postoperative outcomes after elective abdominal surgeries is of interest. Hence, a total of 138 patients were analyzed with 68 receiving ERAS-based care and 70 receiving conventional management. The ERAS group had significantly lower pain scores, reached their recovery milestones sooner and had fewer complications. The average length stay was significantly shorter and there were no increased readmission rates. Thus, we show continued dissemination and implementation of ERAS protocols for improved surgical recovery.

## Background:

Enhanced Recovery after Surgery (ERAS) is a multimodal and evidence-based perioperative care pathway that aims to minimize the physiological stress response to surgery, promote homeostasis and provide a speedy recovery of function [1]. First introduced to colorectal surgery in the 1990s, ERAS protocols have since been widely implemented in several surgical specialties, including hepatobiliary, urologic, gynecologic and thoracic surgery with reproducible effectiveness [2]. ERAS encompasses surgical, anesthetic and nursing care, all standardized in a protocol consistent with individualized care and multidisciplinary team care. Many elements of ERAS include extensive preoperative counseling, no prolonged fasting or preoperative fasting, preoperative carbohydrate loading, multimodal analgesia with minimal opioid use, early removal of drains and catheters, resumption of diet early postoperative and mobilizing patients early [3, 4]. In general, these activities decrease surgical stress response, maintain physiologic function and minimize postoperative morbidity. There are many randomized controlled trials and meta-analyses demonstrating that ERAS reduces length of stay, complications and costs, while improving quality of recovery and patient satisfaction [5, 6]. In spite of some promising findings, there are some obstacles to their wider implementation. Variability in ERAS compliance, resistance to moving away from established practices and safety when performing complex surgeries, is still limiting some areas of implementation [7]. While efficacy has been well established in controlled environments, the effectiveness of ERAS in everyday clinical practices requires on-going validation with real-world studies. These studies will be needed to evaluate feasibility, safety and outcomes in various surgical populations while accounting for the inherent variability and differences in healthcare systems. Therefore, it is of interest to see how the ERAS protocols impact patient outcomes in elective abdominal surgeries- particularly regarding postoperative pain, mobilization, complications, length of stay and recovery milestones as compared to standard perioperative care.

## Materials and Methods:

This retrospective cohort study was conducted at a tertiary care hospital, reviewing the medical records of 138 adult patients who underwent elective abdominal surgeries colorectal, hepatobiliary, or gastric between January 2022 and December 2023. Patients were divided into two groups: the ERAS group (n=68), who were managed using standardized Enhanced Recovery after Surgery protocols and the conventional care group (n=70), who received traditional perioperative management. The ERAS protocol included preoperative patient counseling, carbohydrate loading up to two hours before surgery, avoidance of routine bowel preparation, intraoperative fluid restriction, multimodal analgesia with minimal opioid use, early removal of catheters and drains, early oral feeding and mobilization within 12-24 hours postoperatively. Conventional care followed the institution's previous practices, including prolonged fasting, delayed oral intake, liberal fluid administration, opioid-based pain management and delayed ambulation. Data collected included patient demographics, type of surgery, ASA classification, intraoperative details, postoperative pain scores (VAS), opioid usage, time to ambulation, time to first oral intake and bowel movement, complication rates using Clavien-Dindo classification, duration of hospital stay and 30-day readmission rates. Patients undergoing emergency surgery, those with ASA class IV or higher, or those requiring ICU-level postoperative care were excluded. Data were analyzed using descriptive and inferential statistics to compare outcomes between groups.

## Results:

Patients managed under ERAS protocols demonstrated superior postoperative outcomes compared to those under conventional care. Significant improvements were noted in pain control, earlier mobilization, reduced opioid usage, faster return of bowel function and shorter hospital stay, along with a lower incidence of complications. Table 1 (see PDF) shows baseline characteristics between both groups were similar, ensuring comparability for outcome assessment. Table 2 (see PDF) shows the distribution of surgery types was similar in both groups, allowing for a balanced comparison. Table 3 (see PDF) shows patients in the ERAS group reported significantly lower pain scores at all postoperative intervals. Table 4 (see PDF) shows time to first oral intake was markedly shorter in the ERAS group. Table 5 (see PDF) shows earlier mobilization was observed in ERAS patients compared to conventional care. Table 6 (see PDF) shows that the ERAS protocols significantly reduced the length of hospital stay. Table 7 (see PDF) shows Overall complication rates were lower in the ERAS group, especially for minor complications. Table 8 (see PDF) shows readmission rates within 30 days were lower in the ERAS group, though not statistically significant. Table 9 (see PDF) shows return of bowel function was significantly faster in ERAS patients. Table 10 (see PDF) shows postoperative opioid consumption was significantly lower in ERAS patients.

## Discussion:

The findings of this retrospective cohort study demonstrate that Enhanced Recovery after Surgery (ERAS) protocols significantly improve postoperative outcomes in patients undergoing elective abdominal surgeries. Compared to conventional care, ERAS patients experienced earlier mobilization, faster return of gastrointestinal function, reduced opioid consumption and significantly lower pain scores [6, 7]. The shorter hospital stay observed in the ERAS group not only suggests faster physiological recovery but also has important implications for healthcare resource utilization and cost-effectiveness [8]. Importantly, ERAS patients experienced fewer complications and higher satisfaction levels, indicating a strong alignment between clinical efficacy and patient-centered care [9, 10]. Although the reduction in 30-day readmission was not statistically significant, the trend favored ERAS, supporting its safety in early discharge protocols [11]. These results reinforce the growing body of evidence supporting ERAS implementation as a standard of care across surgical disciplines.

## Conclusion:

ERAS protocols substantially enhance recovery, reduce complications and shorten hospital stay following elective abdominal surgery. Their adoption leads to safer, faster and more patient-friendly postoperative outcomes. Broad integration of ERAS principles should be encouraged across surgical care pathways to optimize both clinical and operational results.

## References

[1] Kannan V (2025). Patient Saf Surg..

[2] Damania R (2017). Ann Transl Med..

[3] Mundhra R (2025). Eur J Obstet Gynecol Reprod Biol X..

[4] Abilashini GD (2025). J Pharm Bioallied Sci..

[5] Özdemir MG (2025). BMC Pregnancy Childbirth..

[6] Jiao S (2024). Eur J Phys Rehabil Med..

[7] Carter-Brooks CM (2018). Am J Obstet Gynecol..

[8] Wang B (2024). Sci Rep..

[9] Kuemmerli C (2022). Br J Surg..

[10] Silverstein J (2023). Obes Surg..

[11] Giannarini G (2019). Minerva Urol Nefrol..

